# The polymorphism and the geographical distribution of the knockdown resistance (*kdr*) of *Anopheles sinensis* in the Republic of Korea

**DOI:** 10.1186/1475-2875-11-151

**Published:** 2012-05-03

**Authors:** Seunghyun Kang, Jongwoo Jung, Sanghui Lee, Heeseung Hwang, Won Kim

**Affiliations:** 1 School of Biological Sciences, Seoul National University, 599 Gwanak-ro, Seoul, Republic of Korea; 2 Department of Science Education, Ewha Womans University, 52 Ewhayeodae-gil, Seoul, Republic of Korea

**Keywords:** *Anopheles sinensis*, Pyrethroid, Knockdown resistance, *Kdr* genotype

## Abstract

**Background:**

In the Republic of Korea (ROK), six sibling species of the *Anopheles sinensis* complex are considered the vector species of malaria, but data on their susceptibilities to malaria and vector capacities have been controversial. The intensive use of insecticides has contributed to the rapid development and spread of insecticide resistance in the *An. sinensis* complex. Knockdown resistance (*kdr*) to pyrethroids and DDT in the *An. sinensis* complex is associated with a mutation in codon 1014 of the voltage-gated sodium channel (VGSC) gene. Because the degree of insecticide resistance varies among mosquito species and populations, the detection of *kdr* mutations among the six sibling species of the *An. sinensis* complex is a prerequisite for establishing effective long-term vector control strategies in the ROK

**Methods:**

In order to investigate species-specific *kdr* mutations, *An. sinensis* complex specimens have been collected from 22 sites in the ROK. Because of the difficulties with species identifications that are based only on morphological characteristics, molecular identification methods have been conducted on every specimen. Part of the IIS6 domain of the VGSC was polymerase chain reaction-amplified and directly sequenced.

**Results:**

The molecular analyses revealed that mutations existed at codon 1014 only in *An. sinensis* sensu stricto and no mutations were found in the other five *Anopheles* species. In *An. sinensis* s.s., one wild type (TTG L1014) and three mutant types (TTT L1014F, TTC L1014F, and TGT L1014C) of *kdr* alleles were detected. The TTC L1014F mutation was observed for the first time in this species.

**Conclusions:**

The fact that the highly polymorphic *kdr* gene is only observed in *An. sinensis* s.s., out of the six *Anopheles* species and their geographical distribution suggest the need for future studies of insecticide resistance monitoring and investigations of species-specific resistance mechanisms in order to build successful malaria vector control programmes in the ROK.

## Background

Before the 1960s, the Republic of Korea (ROK) was a *Plasmodium vivax* malaria-endemic country, and malaria prevailed throughout the country. In the late 1970s, malaria was eliminated through eradication efforts by the World Health Organization and the government of the ROK. However, since 1993, malaria has re-emerged in the north-western region of Gyeonggi-do [1], and over 1,000 cases of malaria have been diagnosed every year with a peak of 4,142 cases diagnosed in 2000 [2]. Although the malaria-vector mosquitoes, the *Anopheles* species, are prevalent throughout the Korean Peninsula, most of the incident cases in the ROK have been reported in the northern part of Gyeonggi-do and the north-western part of Gangwon-do near the Demilitarized Zone (DMZ) between North Korea and the ROK [3]. An area of high transmission is found in North Korea along the DMZ as well, which suggests a parallel outbreak occurred in both countries [4].

In the ROK, eight *Anopheles* species, including the two recently reported species of *Anopheles belenrae* and *Anopheles kleini*, have been recorded so far [5,6]. These eight species belong to three groups (Hyrcanus, Barbirostris, and Lindesayi) in the subgenus *Anopheles*. The Hyrcanus Group consists of 27 species, six in the Lesteri subgroup, four in the Nigerrimus subgroup, and the rest 17 unplaced species within the group [7]. The following six of these species have been reported in the ROK: *Anopheles sinensis* sensu stricto, *Anopheles lesteri, Anopheles pullus, Anopheles sineroides, An. kleini and An. belenrae *[5,6,8,9]. These six species comprise a species complex called *An. sinensis* sensu lato. Because they occur in sympatry and it is hard to distinguish by morphology *An. belenrae* and *An. kleini* from *An. pullus* and *An. sinensis* s.s. [10-14]. Thus, molecular methods have been used for species identification [9,13,15,16]. The other two non-Hyrcanus species, *Anopheles koreicus* and *Anopheles lindesayi*, belong to the Barbirostris and Lindesayi Groups, respectively [6].

The primary vector species for malaria in Korea has long been declared *An. sinensis* s.s. [3,13], but their *P. vivax* malaria susceptibilities and vectorial capacities have been controversial. Recent studies have suggested that *An. pullusAn. kleini* and *An. lesteri* are the primary vector species and that *An. sinensis* s.s. does not play a primary role [2,15,17]. In addition, *An. sineroides* and *An. belenrae* have been reported as *P. vivax*-positive based on enzyme-linked immunosorbent assays and polymerase chain reactions (PCR) [18,19].

Because all six species of *An. sinensis* s.l. in the ROK transmit the malaria parasite *P. vivax*, studying their discriminative ecology, such as their blood feeding and resting behaviours, their larval habitats, and their responses to insecticides, is necessary for malaria control. Hybridization between related species of the anopheline species, which rarely takes place in natural environments, might complicate malaria vector control [20-23]. Evidence of introgressive hybridization has been reported for the *Anopheles gambiae* complex [20-26]. In addition, recent studies have reported the presence of natural hybrids between some anopheline species in the ROK [27].

Until now, the use of insecticides has been the most effective and economical vector control method for malaria and other vector-borne diseases, such as dengue and filariasis [28]. Since the first introduction of insecticides to the ROK in the 1970s, pyrethroids and organophosphates have been used throughout the country in order to control medically and agriculturally important arthropod pests, including mosquitoes. The national malaria eradication programme that was based mostly on the chemical control of vector mosquitoes was successful in the 1990s in the ROK. However, overdoses of insecticide have quickly led to the presence and spread of insecticide-resistant mosquitoes [13], which have caused serious problems for malaria-controlling interventions.

Resistance to pyrethroids and DDT, which is known as knockdown resistance (*kdr*), is caused by a single mutation in the S6 transmembrane segment of domain II in the voltage-gated sodium channel (VGSC) gene [29]. Several mutations at codon 1014, such as L1014F (Leu-to-Phe), L1014S (Leu-to-Ser), and L1014C (Leu-to-Cys) have been reported in many *Anopheles* species, including *An. gambiae, Anopheles arabiensis, Anopheles culicifacies, Anopheles stephensi, An. sinensis* complex, *Anopheles sacharovi, Anopheles subpictus, Anopheles sundaicus, Anopheles aconitus, and Anopheles vagus *[30-37]. A positive correlation between the *kdr* genotype and the resistance phenotype to pyrethroids and DDT in the *Anopheles* species was well documented by articles [34,38,39].

A previous study reported that the frequencies of the *kdr* allele of *An. sinensis* in the ROK ranged from 25.0 to 96.6%, which suggested that pyrethroid resistance was already widespread in natural populations of the ROK [34]. A standard World Health Organization insecticide susceptibility test was conducted on the *Anopheles* species in the ROK [40-42], and the results showed the development and widespread distribution of pyrethroid-resistant phenotypes. In order to monitor knockdown mutations of pyrethroid resistance in *An. sinensis*, real-time PCR amplification of a specific allele (rtPASA) has been developed. The results suggested that L1014F mutation was a major allele that showed a high allele frequency, whereas L1014C mutation was a minor allele that showed a low allele frequency within the *An. sinensis* populations in the ROK [34]. However, these previous studies conducted chemical and molecular assays on the *An. sinensis* group and species-specific assays have never been conducted because the sibling species are morphologically indistinguishable, and, unlike *An. sinensis* s.s., the other species have relatively low population densities. Because the mechanisms and degree of insecticide resistance vary among species and populations [43-46], the present study aimed to explore the species-specific distribution of *kdr* resistance alleles in all members of the *An. sinensis* complex in the ROK.

## Methods

### Mosquito collections and species identification

**Figure 1:**
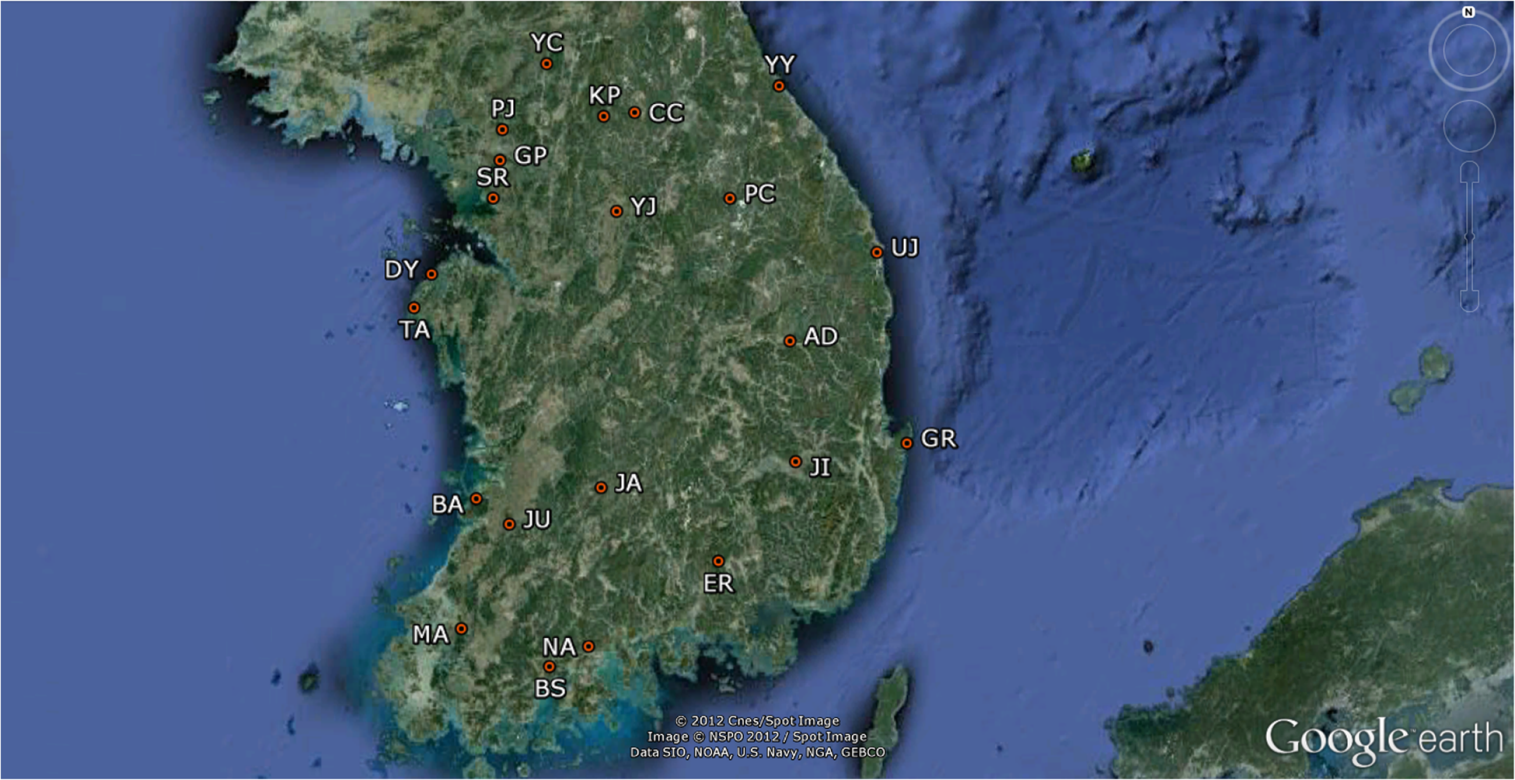
The geographical locations of the sampling sites in a map of the Republic of Korea (ROK).

Mosquitoes were collected from 22 locations (Figure 1, Table 1) in the ROK with a CDC Miniature Light Trap (John W Hock Company, Gainesville, FL, USA) and preserved on site using dry ice. Because it is morphologically hard to distinguish the species within the *An. sinensis* complex, the mosquito specimens were isolated based on their morphological characteristics that were determined first under a stereomicroscope in the laboratory compared to the other insects, and then the specific species of all of the *Anopheles* specimens were identified using multiplex assays [47]. DNA was extracted from the entire body or legs by following a standard phenol extraction protocol or DNeasy Blood and Tissue kit (QIAGEN, USA).

**Table 1:** Summary of regional information and the species composition of the *An. sinensis* complex

**Name**	**Sample Sites**	**N**	**Identified Species**					
			***An. sinensis***	***An. pullus***	***An. kleini***	***An. belenrae***	***An. lesteri***	***An. sineroeides***
AD	ANDONG	16	16	0	0	0	0	0
BA	BUAN	16	16	0	0	0	0	0
BS	BOSEONG	13	13	0	0	0	0	0
CC	CHOONCHEON	8	8	0	0	0	0	0
DY	DANYANG	15	15	0	0	0	0	0
ER	EUIRYEONG	25	25	0	0	0	0	0
GP	GIMPO	237	208	10	5	2	12	0
GR	GURYONGPO	17	17	0	0	0	0	0
JA	JINAN	11	11	0	0	0	0	0
JI	JAIN	29	29	0	0	0	0	0
JU	JEONGEUP	10	10	0	0	0	0	0
KP	KAPYEONG	14	14	0	0	0	0	0
MA	MOOAN	6	6	0	0	0	0	0
NA	NAKAN	28	28	0	0	0	0	0
PC	PYEONGCHANG	8	0	0	8	0	0	0
PJ	PAJOO	127	114	13	0	0	0	0
SR	SORAE	25	24	0	0	0	1	0
TA	TAEAN	12	12	0	0	0	0	0
UJ	ULJIN	16	16	0	0	0	0	0
YC	YEONCHEON	80	62	3	15	0	0	0
YJ	YEOJOO	12	11	0	1	0	0	0
YY	YANGYANG	30	10	0	n	0	0	19
		**755**	**665**	**26**	**30**	**2**	**13**	**19**

### DNA sequencing of VGSC

In order to detect the *kdr* mutation, part of the IIS6 domain of the VGSC was PCR amplified and directly sequenced using 5′ASIIS56 and 3′ASIIS56 intron primers [34]. The sequences were determined using an ABI 3730xl DNA analyser (Applied Biosystems, USA) and visually confirmed using Sequence Navigator 1.1 software (Applied Biosystems, USA). The obtained sequences were aligned with Clustal X version 2.0 [48]. The deduced amino acid comparisons were conducted by MEGA version 5 [49].

### Statistical analyses

The maximum likely frequency (y) of an allele present or absent in a sample of a given size (x) was obtained from the upper of 95% confidence limit of binomial distribution, given by y = 1-0.05^1/x^, following the example of Post and Millest [50].

## Results

### Species composition and distribution

A total of 755 *An. sinensis* complex mosquitoes were collected from 22 sites in the ROK, as briefly described in Figure 1 and Table 1. As a result of the molecular identifications that were conducted by multiplex assays (Table 1), most of the specimens were *An. sinensis* s.s. (665 of 755, 88.08%), which is known as the dominant species in the ROK, and these were followed by *An. kleini* (30 of 755, 3.97%), *An. pullus* (26 of 755, 3.44%), *An. sineroides* (19 of 755, 2.52%), *An. lesteri* (13 of 755, 1.72%), and *An. belenrae* (two of 755, 0.26%). Hybrid individuals were not detected. *An. sinensis* s.s. was found at most of the sites (21 of 22), whereas the other species were found at a few sites (one to five of 22) in the northern part of the ROK, and these results were in concordance with previous studies showing topoclinal distributions.

### *kdr* mutations

**Figure 2:**
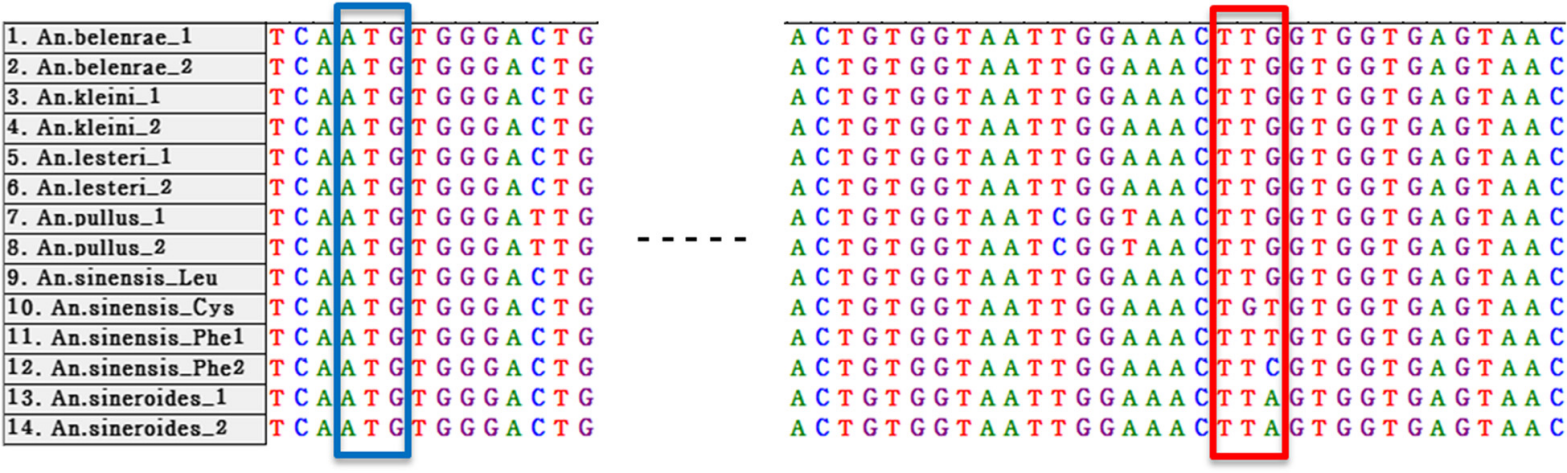
**Alignment of multiple DNA sequences at codon 918 and codon 1014 within the voltage-gated sodium channel gene in the *An. sinensis* complex in the ROK.** The blue box indicates codon 918 and the red box indicates codon 1014.

In order to examine the mutations at codon 1014, 343-bp sequences of part of the IIS6 domain of the VGSC gene were obtained from a total of 177 specimens that consisted of *An. sinensis* s.s. (n = 87), *An. kleini* (n = 30), *An. pullus* (n = 26), *An. belenrae* (n = 2), *An. lesteri* (n = 13), and *An. sineroides* (n = 19) from 10 sites in the ROK (Table 2 and 3). The DNA sequences were different among the species. However, the deduced amino acid sequences showed no non-synonymous mutations within the VGSC-coding regions in all six species, except for at codon 1014. Pyrethroid- and DDT-resistant *kdr* mutations were detected in *An. sinensis* s.s. only, and the other five species had the wild-type *kdr* allele L1014 only (Figure 2, Table 2 and 3). Interestingly, *An. sineroides* had a different amino-acid coding sequence (TTA). But the same amino acid (leucine) occurred at codon 1014. Met-Thr mutation at codon 918 (M918T), or super-*kdr,* which enhances the resistance in combination with L1014F within the same genetic region was not detected in any of the six species.

**Table 2:** Species-specific genotype frequencies of the *kdr* allele in each study site

**Study Sites**	**Species**	**N**	**Genotype Frequency (%)**									
			**L/L**	L*/L*	**L/P**	**L/C**	**P/P**	**P/C**	**C/C**	L/P*	P/P*	C/P*
YC	*An. sinensis*	14	14.3	0.0	14.3	7.1	28.6	35.7	0.0	0.0	0.0	0.0
	*An. kleini*	15	100.0	0.0	0.0	0.0	0.0	0.0	0.0	0.0	0.0	0.0
	*An. pullus*	3	100.0	0.0	0.0	0.0	0.0	0.0	0.0	0.0	0.0	0.0
PJ	*An. sinensis*	17	11.8	0.0	17.6	0.0	41.2	23.5	5.9	0.0	0.0	0.0
	*An. pullus*	13	100.0	0.0	0.0	0.0	0.0	0.0	0.0	0.0	0.0	0.0
GP	*An. kleini*	5	100.0	0.0	0.0	0.0	0.0	0.0	0.0	0.0	0.0	0.0
	*An. lesteri*	12	100.0	0.0	0.0	0.0	0.0	0.0	0.0	0.0	0.0	0.0
	*An. belenrae*	2	100.0	0.0	0.0	0.0	0.0	0.0	0.0	0.0	0.0	0.0
	*An. pullus*	10	100.0	0.0	0.0	0.0	0.0	0.0	0.0	0.0	0.0	0.0
PC	*An. kleini*	8	100.0	0.0	0.0	0.0	0.0	0.0	0.0	0.0	0.0	0.0
YJ	*An. kleini*	1	100.0	0.0	0.0	0.0	0.0	0.0	0.0	0.0	0.0	0.0
BS	*An. sinensis*	13	0.0	0.0	15.4	0.0	46.2	30.8	7.7	0.0	0.0	0.0
SR	*An. lesteri*	1	100.0	0.0	0.0	0.0	0.0	0.0	0.0	0.0	0.0	0.0
AD	*An. sinensis*	16	50.0	0.0	0.0	31.3	0.0	0.0	0.0	18.8	0.0	0.0
GR	*An. sinensis*	17	58.9	0.0	11.8	0.0	0.0	0.0	0.0	0.0	23.5	5.9
YY	*An. sinensis*	10	0.0	0.0	30.0	0.0	60.0	0.0	0.0	10.0	0.0	0.0
	*An. kleini*	1	100.0	0.0	0.0	0.0	0.0	0.0	0.0	0.0	0.0	0.0
	*An. sineroides*	19	0.0	100.0	0.0	0.0	0.0	0.0	0.0	0.0	0.0	0.0
		**177**										

L indicates the susceptible allele L1014 (TTG), and L* indicates the other susceptible allele L1014 (TTA). P indicates the resistance allele L1014F (TTT), and P* indicates the other codon type allele L1014F (TTC). C indicates the resistance allele L1014C (TGT).

**Table 3:** Species-specific allele frequencies of the *kdr* allele (and 95% confidence intervals, Cls) in each study site

			**kdr Allele Frequency (%)**							
			**Susceptible**		**Resistant**					
**Study Sites**	**Species**	**N**	**Leu**		**Phe**				**Cys**	
			**TTG**	**[95% CI]**	**TTT**	**[95% CI]**	**TTC**	**[95% CI]**	**TGT**	**[95% CI]**
YC	*An. sinensis*	14	25.0	[20.2-29.8]	54.0	[43.6-64.4]	0.0	[0.0-0.0]	21.0	[17.0-25.0]
	*An. kleini*	15	100.0	[81.9-100.0]	0.0	[0.0-0.0]	0.0	[0.0-0.0]	0.0	[0.0-0.0]
	*An. pullus*	3	100.0	[36.8-100.0]	0.0	[0.0-0.0]	0.0	[0.0-0.0]	0.0	[0.0-0.0]
PJ	*An. sinensis*	17	20.6	[17.3-23.9]	61.8	[51.8-71.7]	0.0	[0.0-0.0]	17.6	[14.8-20.5]
	*An. pullus*	13	100.0	[79.4-100.0]	0.0	[0.0-0.0]	0.0	[0.0-0.0]	0.0	[0.0-0.0]
GP	*An. kleini*	5	100.0	[54.9-100.0]	0.0	[0.0-0.0]	0.0	[0.0-0.0]	0.0	[0.0-0.0]
	*An. lesteri*	12	100.0	[77.9-100.0]	0.0	[0.0-0.0]	0.0	[0.0-0.0]	0.0	[0.0-0.0]
	*An. belenrae*	2	100.0	[22.4-100.0]	0.0	[0.0-0.0]	0.0	[0.0-0.0]	0.0	[0.0-0.0]
	*An. pullus*	10	100.0	[74.1-100.0]	0.0	[0.0-0.0]	0.0	[0.0-0.0]	0.0	[0.0-0.0]
PC	*An. kleini*	8	100.0	[68.8-100.0]	0.0	[0.0-0.0]	0.0	[0.0-0.0]	0.0	[0.0-0.0]
YJ	*An. kleini*	1	100.0	[5.0-100.0]	0.0	[0.0-0.0]	0.0	[0.0-0.0]	0.0	[0.0-0.0]
BS	*An. sinensis*	13	7.7	[6.1-9.3]	69.2	[55.0-83.5]	0.0	[0.0-0.0]	23.1	[18.3-27.8]
SR	*An. lesteri*	1	100.0	[5.0-100.0]	0.0	[0.0-0.0]	0.0	[0.0-0.0]	0.0	[0.0-0.0]
AD	*An. sinensis*	16	75.0	[62.2-87.8]	0.0	[0.0-0.0]	9.0	[7.5-10.5]	16.0	[13.3-18.7]
GR	*An. sinensis*	17	64.7	[54.3-75.2]	17.6	[14.8-20.5]	14.7	[12.3-17.1]	2.9	[2.5-3.4]
YY	*An. sinensis*	10	23.5	[17.4-29.6]	70.6	[52.3-88.9]	5.9	[4.4-7.4]	0.0	[0.0-0.0]
	*An. kleini*	1	100.0	[5.0-100.0]	0.0	[0.0-0.0]	0.0	[0.0-0.0]	0.0	[0.0-0.0]
	*An. sineroides*	19	100.0*	[85.4-100.0]	0.0	[0.0-0.0]	0.0	[0.0-0.0]	0.0	[0.0-0.0]
		**177**								

The asterisk indicates TTA L1014 allele frequency.

### *An. sinensis* s.s.

A total of four types, which consisted of one wild type (TTG L1014) and three mutant types (TTT L1014F, TTC L1014F, and TGT L1014C), of *kdr* alleles were detected in *An. sinensis* s.s.. TTC L1014F was found for the first time. TTT L1014F was the dominant mutation in all of the regions, whereas TTC L1014F, which was distributed in the eastern part of the ROK alongside the Taebaek Mountain (YY, AD, and GR in Figure 1 and Table 1 and 2), existed only in the heterozygotic state (Table 2). The allele frequencies of L1014 ranged from 7.7% to 75.0%, while that of L1014F (TTT) ranged from 0% to 70.6%, that of L1014F (TTC) ranged from 0% to 14.7%, and that of L1014C ranged from 0% to 23.1% (Table 3).

## Discussion

Knockdown resistance (*kdr*) is caused by mutations at codon 1014 of the VGSC, which is the target of pyrethroids and DDT. In this study, molecular analysis of the VGSC in the *An. sinensis* complex in the ROK revealed that mutations at codon 1014 existed only in *An. sinensis* s.s., whereas no *kdr* mutations were observed in the other five species, *including An. pullus, An. kleini, An. sineroides, An. lesteri* and *An. belenrae*. For wild-type L1014, a previous comparative study of the VGSC of various taxa showed different usages of coding triplets at L1014 [51], while in the present results, the amino-acid coding sequences at L1014 differed between the species. The codon of L1014 was TTC in five species (*An. sinensis* s.s., *An. pullus, An. kleini, An. lesteri* and *An. belenrae*), whereas for *An. sineroides* it was TTA, which is observed in a number of species, including *An. gambiae An. arabiensis* and *An. vagus *[30,36,37].

Pyrethroids have been widely used in large quantities for the control of agricultural pests and medically important arthropod pests through thermal fogging, residual spraying, impregnated clothing, and mosquito nets since their introduction to the ROK in the 1970s. Inevitably, a variety of mosquitoes, including malaria vectors, might have developed resistance to pyrethroids and jeopardized the successful malaria control programmes in the ROK [34,40,42]. A number of recent studies have reviewed the numerous cases that were examined and whether there were correlations between mutations in the VGSC and the resistance phenotype in diverse taxa [38,51,52]. Almost all of the results of the previous studies have shown a strong causal relationship between the *kdr* genotype and insecticide susceptibility to pyrethroids and DDT. However, the absence of a *kdr* mutation is not a causal factor of total susceptibility to the insecticides.

Although the detailed research on fitness costs of *kdr* mutations, additional biochemical assays and specific gene expression studies are needed for future study, the highly polymorphic *kdr* gene that was observed only in *An. sinensis* s.s. and the absence of a *kdr* mutation in the other five species suggest two tentative hypotheses. First, because the anopheline species other than *An. sinensis* s.s. are geographically distributed in the northern part of the ROK near the DMZ, the absence of *kdr* mutations in these species may have resulted from migration of these *kdr*-free mosquitoes from North Korea or the DMZ to the ROK to seek for blood sources, such as cows or pigs. Along the DMZ and in North Korea, the environment was unsuitable for the mosquitoes due to the lack of blood sources. However, the amount of insecticide used in North Korea and along the DMZ was much less than the amount used in the ROK, which reduces the insecticide selection pressure [53]. This hypothesis is in concordance with the renowned hypothesis that the re-emergence of *P. vivax* in the ROK arose from North Korea through sporozoite-infected mosquitoes that dispersed from North Korea to the ROK across the DMZ [3,53-57]. Second, the highly polymorphic *kdr* gene in *An. sinensis* s.s. (the dominant *Anopheles* mosquito in East Asia) in the ROK may be explained by their large population size and wide species range. The level of genetic variation within a species is generated by mutation and eliminated by genetic drift due to finite population size [58]. Large population size and wide species range are precondition to polymorphism, as a large effective population size will have an increased genetic variability [59,60] and a higher rate of mutation [61].

For *An. sinensis* s.s., the geographical distribution of *kdr* alleles is supported by a previous study that examined the population genetic structure of *An. sinensis* using mitochondrial control regions, and the results suggested distinct subdivisions in the Northern Group (NG) and Southern Group (SG) in the ROK [62]. The Great Mountains, such as the Sobaek and Taebaek Mountains that cross the ROK from the northeast to southwest may play a major role as potent genetic barriers. These two groups have different genetic properties, such that the SG is genetically more diverse and has a larger number of private alleles and effective population size than the NG. A total of four *kdr* alleles including one wild type and three mutant types were observed in this study. The three types of alleles (TTG L1014, TTT L1014F, and TGT L1014C) were observed in both the NG and the SG. Whereas the TTC L1014F allele, which was the rarest allele and which was detected for the first time in *An. sinensis* s.s. in this study, was only detected in the SG (AD, GR, and YY in Figure 1 and Table 2 and 3).

In summary, the different allele statuses of *kdr* genes in the *An. sinensis* complex suggest the importance of monitoring insecticide susceptibilities and the resistance of target vectors in control programmes. Effective resistance monitoring that is based on species-specific insecticide bioassay tests, molecular studies of allele diversity, origins of insecticide resistance, and minor resistance mechanisms (behavioural and cuticular resistance) will be crucial for building successful malaria vector control programmes that can explain and predict the development and spread of insecticide resistance traits.

## Conclusions

This is the first report that has explored the presence and absence of 1014 codon mutations in the VGSC of the six malaria-vector species in the ROK. The highly polymorphic 1014 mutations were only observed in *An. sinensis* s.s., while the other five species showed no mutations. In *An. sinensis* s.s., the rarest allele, L1014F (TTC), was detected for the first time in this study, and the distribution of this allele was restricted to the southeast of the ROK. The present study is the first step in exploring species-specific insecticide resistance mechanisms. Further in-depth experiments are needed to prove the current epidemiological and evolutionary dynamics of malaria vectors in the ROK. In addition, these efforts will be one of the prerequisite steps needed to establish effective long-term vector control strategies in the ROK.

## Competing interests

The authors declare that they have no competing interests.

## Authors’ contributions

SK performed the molecular assays, data analysis, and manuscript writing. JJ collected field samples and identified the mosquitoes based on their morphological characteristics. SL and HH identified the mosquitoes by molecular identification methods. JJ and WK supervised the work and manuscript writing. WK was responsible for the management of and fund raising for this study. All authors read and approved the final manuscript.
